# Randomised phase III trial of carboplatin plus etoposide *vs* split doses of cisplatin plus etoposide in elderly or poor-risk patients with extensive disease small-cell lung cancer: JCOG 9702

**DOI:** 10.1038/sj.bjc.6603810

**Published:** 2007-06-19

**Authors:** H Okamoto, K Watanabe, H Kunikane, A Yokoyama, S Kudoh, T Asakawa, T Shibata, H Kunitoh, T Tamura, N Saijo

**Affiliations:** 1 Department of Respiratory Medicine, Yokohama Municipal Citizen's Hospital, 56 Okazawa-cho, Hodogaya-ku, Yokohama, Kanagawa 240-8555, Japan; 2 Niigata Cancer Center Hospital, Niigata-city, Japan; 3 Osaka City University Medical School, Osaka-city, Japan; 4 National Cancer Center, Tokyo, Japan; 5 National Cancer Center Hospital, Tokyo, Japan; 6 National Cancer Center East Hospital, Kashiwa, Japan

**Keywords:** small-cell lung cancer, carboplatin, cisplatin, etoposide, elderly, poor-risk

## Abstract

We compared the efficacy and the safety of a carboplatin plus etoposide regimen (CE) *vs* split doses of cisplatin plus etoposide (SPE) in elderly or poor-risk patients with extensive disease small-cell lung cancer (ED-SCLC). Eligibility criteria included: untreated ED-SCLC; age ⩾70 and performance status 0–2, or age <70 and PS 3. The CE arm received carboplatin area under the curve of five intravenously (IV) on day 1 and etoposide 80 mg m^−2^ IV on days 1–3. The SPE arm received cisplatin 25 mg m^−2^ IV on days 1–3 and etoposide 80 mg m^−2^ IV on days 1–3. Both regimens were given with granulocyte colony-stimulating factor support in a 21–28 day cycle for four courses. A total of 220 patients were randomised. Median age was 74 years and 74% had a PS of 0 or 1. Major grade 3–4 toxicities were (%CE/%SPE): leucopenia 54/51, neutropenia 95/90, thrombocytopenia 56/16, infection 7/6. There was no significant difference (CE/SPE) in the response rate (73/73%) and overall survival (median 10.6/9.9 mo; *P*=0.54). Palliation scores were very similar between the arms. Although the SPE regimen is still considered to be the standard treatment in elderly or poor-risk patients with ED-SCLC, the CE regimen can be an alternative for this population considering the risk–benefit balance.

Approximately half of patients with small-cell lung cancer (SCLC) are older than 70 years, and the proportion of elderly SCLC patients is continuously increasing in Japan (Morita, 2002). However, since many investigators have arbitrarily excluded elderly patients from clinical trials, no standard chemotherapeutic regimen has been established for elderly patients with SCLC. The Japan Clinical Oncology Group (JCOG) has reported that carboplatin plus etoposide (CE) is an active and less toxic regimen in elderly patients with SCLC (Okamoto *et al*, 1999). However, other clinical trials have indicated that the combination chemotherapy of reduced (Souhami *et al*, 1997) or split doses of cisplatin plus etoposide (SPE) (Murray *et al*, 1998; Westeel *et al*, 1998) can be safely and effectively administered in elderly or poor-risk patients with SCLC. Therefore, we conducted a phase III trial comparing CE with SPE in elderly or poor-risk patients with SCLC. Although elderly is not the same as poor-risk, many clinical trials for the elderly have included both types of patients. Therefore, we decided to include both elderly and poor-risk patients with SCLC at the time of proposal for this phase III trial.

## PATIENTS AND METHODS

### Patient selection

Eligibility criteria included patients with histologically or cytologically confirmed SCLC who were ⩾70 years of age and had an Eastern Cooperative Oncology Group performance status (PS) of 0–2, or who were <70 years in age and had a PS of 3. Additional criteria consisted of extensive disease (ED), chemotherapy-naive, evaluable or measurable disease, expected survival ⩾2 months, adequate organ functions (leucocyte count ⩾4000 mm^−3^, platelet count ⩾100 000 mm^−3^, haemoglobin level ⩾9.0 g dl^−1^, AST/ALT ⩽2 × upper limit of normal range, total bilirubin ⩽1.5 mg dl^−1^, creatinine ⩽1.5 mg dl^−1^, 24-h creatinine clearance (Ccr) ⩾50 ml min^−1^, and PaO2 ⩾60 mmHg), no symptomatic pericardial or pleural effusion requiring drainage, no active concomitant malignancy, no senile dementia, and written informed consent. Exclusion criteria included brain metastases requiring radiotherapy, superior vena cava (SVC) syndrome requiring radiotherapy, serious medical or psychiatric illness, or pregnancy or lactation. Staging procedures included chest X-ray, computed tomography (CT) scan of the chest, CT scan or magnetic resonance imaging (MRI) of the brain, CT scan or ultrasound of the abdomen, isotope bone scanning, and bone marrow aspiration or biopsy.

### Treatment protocol

Patients were randomised to either the CE arm or the SPE arm. The CE regimen consisted of carboplatin area under the curve (AUC) of five intravenously (IV) on day 1 and etoposide 80 mg m^−2^ IV on days 1, 2, and 3. The SPE regimen consisted of cisplatin 25 mg m^−2^ IV on days 1, 2, and 3 and etoposide 80 mg m^−2^ IV on days 1, 2, and 3. Cycles were repeated every 3–4 weeks for up to four courses. In our previous phase II study using the CE regimen for elderly patients with SCLC, carboplatin AUC of 5 on day 1 and etoposide 100 mg m^−2^ on days 1, 2, and 3 were administered every 4 weeks (Okamoto *et al*, 1999). However, because grade 3 or 4 neutropenia occurred in 91% of the patients, in the current phase III trial we decided to reduce the etoposide dosage to 80 mg m^−2^ on days 1, 2, and 3, and repeat the cycle every 3–4 weeks instead of every 4 weeks. Twenty-four-hour Ccr was substituted for glomerular filtration rate (GFR) in Calvert’s formula. Antiemetic prophylaxis with 5-HT3 antagonists plus dexamethasone was used at the treating physician’s discretion. According to the Japanese approved guideline, prophylactic use of recombinant human granulocyte colony-stimulating factor (G-CSF) was recommended for daily administration after day 4 until the leucocyte (neutrophil) count exceeded 10 000 (5000) mm^−3^. If the leucocyte (neutrophil) count decreased to less than 3000 (1500) mm^−3^, then G-CSF was restarted. However, the actual use of G-CSF was left at the discretion of the treating physician. Subsequent courses of chemotherapy were initiated when leucocyte count ⩾3000 mm^−3^; platelet count ⩾75 000 mm^−3^; Cr⩽1.5 mg dl^−1^; AST/ALT⩽2.5 × upper limit of normal range; and either PS⩽2 and age⩾70 years, or PS⩽3 and age<70 years were satisfied both after day 21 and two or more days after the discontinuation of G-CSF. If the above criteria were not satisfied by the first day of the next course, treatment was withheld until full recovery. If more than 6 weeks passed from day 1 of the last course, the patient was removed from protocol treatment. Dose modifications were made based only on grade 4 haematologic toxicities. If grade 4 leucopenia or neutropenia lasting 4 days or more was present, or grade 4 thrombocytopenia occurred, the doses for the next course were carboplatin AUC of 4 on day 1, cisplatin 20 mg m^−2^ for 3 days, and etoposide 60 mg m^−2^ for 3 days. If the same haematologic toxicity was observed after dose reduction, the patient was removed from protocol treatment. If grade 3 or 4 non-haematologic toxicities, except for nausea/vomiting and hyponatraemia, occurred, the patient was removed from protocol treatment even if the toxicities improved thereafter.

Responders after four courses were not allowed to receive further chemotherapy until progressive disease (PD) developed. Although post-protocol treatment was left at the discretion of the physician, crossover treatment was prohibited.

### Evaluation

Tumour responses were evaluated according to World Health Organization criteria (World Health Organization, 1979). Toxicities were evaluated according to JCOG Toxicity Criteria (Tobinai *et al*, 1993), which are similar to the National Cancer Institute-Common Toxicity Criteria (NCI-CTC ver 1) for the grading of toxicities.

### Palliation score

Study-specific eight-item palliation scores were completed by patients before treatment and 3 weeks after the third course of chemotherapy. The attending physicians were not allowed to complete the scores. The items consisted of cough, pain, anorexia, shortness of breath, well-being, nausea, diarrhoea or constipation, and sleep. The items were scored as not at all present (0), a little (1), moderate (2), and very much (3). The sum of the total score for all eight items was compared between the baseline and post-treatment assessments. If the post-treatment score was below the baseline score, the palliation score for that patient was judged as having shown improvement.

### Study design and statistics

This trial was designed as a multicentre, prospective, randomised phase III trial. The study protocol was approved by the Clinical Trial Review Committee of JCOG and the institutional review board of each participating institution before the initiation of the study. The primary endpoint was overall survival (OS). In this study, the experimental arm was the CE arm and the control was the SPE arm. The MST of our previous phase II trial for elderly patients with extensive disease small-cell lung cancer (ED-SCLC) using the CE regimen was 10.1 months. The MST of the SPE regimen for a similar population was not available at the time of the study proposal. Although Westeel and co-workers in 1998 and Murray and co-workers in 1998 reported an excellent MST of SPE plus concurrent chest radiotherapy for elderly or frail patients with limited disease (LD)-SCLC, an MST of the SPE regimen for elderly or frail patients with ED-SCLC was not available at that time. The only data available on the CAV/PE regimen for elderly or poor-risk patients with SCLC using reduced cisplatin (60 mg m^−2^ IV on day 1) were reported by Souhami and co-workers in 1997 and the MST of that study was 5.9 months. Therefore, for statistical calculations in the current phase III trial, we used the MST value of the Souhami trial for the control arm instead of the MST of the SPE regimen. In addition, an individualised AUC-based dosing strategy of carboplatin was expected to have greater efficacy and less toxicity compared with the SPE regimen at that time. This trial was designed as a superiority trial and the planned sample size was 110 patients in each arm for 80% power to detect a 0.67 hazard ratio for CE to SPE in OS at an alpha of 0.025 (one sided) (Schoenfeld and Richter, 1982). Patients were randomised to receive either CE or SPE with a minimisation method for balancing centre, PS (0–1 *vs* 2–3) and age (⩾70 years *vs* <70 years).

Survival distributions were compared by unstratified log-rank test. Proportion of improvement in palliation score was evaluated by Fisher's exact test. The change in each symptom score by treatment arm was evaluated by the Wilcoxon rank-sum test. The relationship between the interval of each chemotherapy course and the two regimens was evaluated by the Wilcoxon rank-sum test. Multivariate analysis was performed using Cox's proportional hazards model to evaluate the importance of seven clinically selected variables (treatment arm, PS, age, sex, lactate dehydrogenase level, alkaline phosphatase level, and leucocyte count) as prognostic factors. All *P*-values in this report are two sided, excluding *P*-values for OS and progression-free survival (PFS).

The interim analysis was performed after half of the planned number of patients had been enrolled in March 2002, with adjustment for multiplicity by the alpha-spending function (DeMets and Lan, 1994) with an O’Brien-Fleming type boundary. Because the interim analysis did not meet the prespecified stopping criteria, the study was continued and the planned accrual of 220 patients was randomised in this trial.

## RESULTS

### Patient characteristics

**Figure 1:**
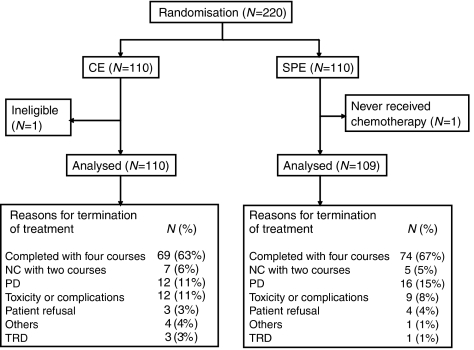
Flow diagram of randomised phase III trial of CE *vs* SPE in elderly or poor-risk patients with extensive disease SCLC.

Between August 1998 and February 2004, a total of 220 patients were registered from 24 institutions. Baseline characteristics were well balanced between the arms. Median age was 74 years, 92% were 70 years or older, 88% were male, and 74% had a PS of 0 or 1 (Table 1). One patient in the CE arm was found to have LD after the completion of protocol chemotherapy due to protocol violation, and this patient was considered ineligible (Figure 1).

### Delivery of treatment

Reasons for termination of treatment are listed in Figure 1, and there were no major differences between the arms. Of the patients, 63% in the CE arm and 67% in the SPE arm completed four courses, and 11% in the CE arm and 8% in the SPE arm did not complete treatment because of toxicity or complications. Treatment-related death (TRD) occurred in four patients; three patients in the CE arm and one in the SPE arm. All TRDs of patients who were ⩾70 years old with a good pretreatment PS (all PS 1) were associated with neutropenic infection, which occurred after the first course of chemotherapy. Although the median interval of chemotherapy was slightly more prolonged in the CE arm than in the SPE arm, total delivered courses were similar between the arms (Table 2). One patient in the SPE arm never received chemotherapy due to the occurrence of delirium after registration. Dose reduction was more frequently observed in the CE arm than in the SPE arm: 29% *vs* 10%, *P*<0.01. Course delay, G-CSF delivery and total courses with G-CSF delivery were similar between the arms.

### Toxicity and palliation score

Toxicities are listed in Table 3. Grade 3 or 4 leucopenia and neutropenia occurred in 54 and 95% of the CE arm *vs* 51 and 90% of the SPE arm, respectively. Grade 3 or 4 thrombocytopenia occurred more frequently in the CE arm than in the SPE arm: 56 *vs* 16%, *P*<0.01. Gastrointestinal toxicities including nausea or vomiting and diarrhoea were mild in both arms. There were few grade 3 or 4 toxicities and no remarkable differences between the arms. Other non-haematologic toxicities were similarly distributed between the arms. Grade 3–4 hyponatraemia, mainly caused by syndrome of inappropriate antidiuretic hormone (SIADH) secretion, occurred in 14–16% of the patients. More importantly, thrombocytopenia occurred more frequently in the CE arm, but none of the patients in either arm showed grade 3 or 4 bleeding. Only one patient in the CE arm showed grade 2 bleeding. Because no grading of febrile neutropenia was listed in JCOG toxicity criteria, the rate of the toxicity was not investigated in this study.

Baseline and post-treatment palliation scores were evaluated in 220/220 (100%) and 208/220 (95%) patients, respectively. We handled missing values by imputing the worst score. Improvement was achieved in 69 (63%) patients in the CE arm *vs* 61 (56%) patients in the SPE arm, although the difference was not statistically significant (*P*=0.34). Similarly, there were no statistical differences in the change of each symptom score between the arms (Table 4).

### Objective tumour response, PFS and OS

**Figure 2:**
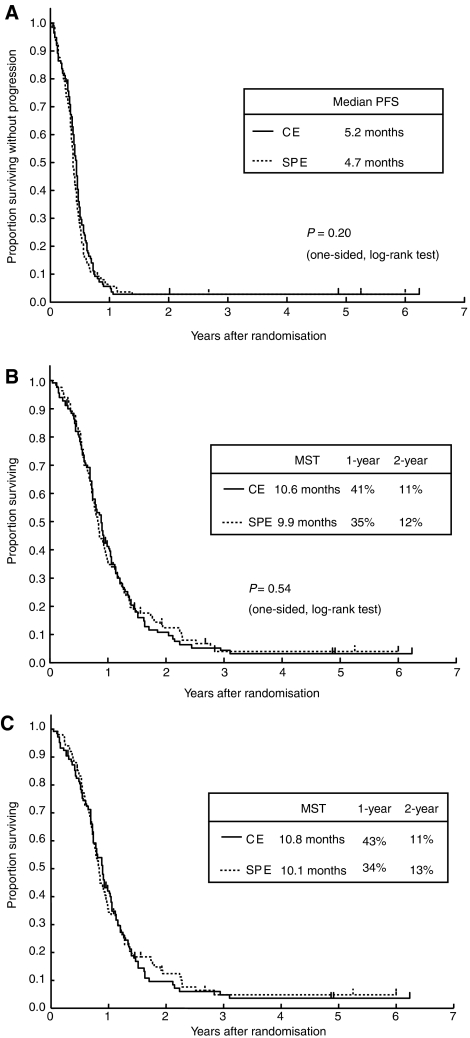
(**A**) PFS curves (*n*=220). (**B**) OS curves (*n*=220). (**C**) Survival curves of the patients ⩾70 years of age with a PS of 0–2 (*n*=202).

The objective response rate of 73% was quite similar between the arms. Five CRs and 75 PRs were observed in each arm (Table 5). Progression-free survival curves and OS curves are shown in Figure 2A and B. Ninety-seven percent of the patients had progressed or died at the time of final analysis. Progression-free survival was quite similar between the arms (*P*=0.20, one sided). The MST was 5.2 months in the CE arm *vs* 4.7 months in the SPE arm. OS was very similar between the arms (*P*=0.54, one sided). The MST and 1-year survival rate was 10.6 months and 41% in the CE arm *vs* 9.9 months and 35% in the SPE arm.

### Second-line chemotherapy

According to an *ad-hoc* survey (not pre-specified in the protocol), 130 (59%) patients (68 (62%) patients in the CE arm and 62 (56%) in the SPE arm) received second-line chemotherapy after relapse and the regimens were almost equally distributed between the arms. The same regimen as the initial chemotherapy, platinum-based combinations, and irinotecan regimens with or without other agents were administered in 17 (15%), 48 (44%), and 40 (36%) patients in the CE arm *vs* 10 (9%), 44 (40%), and 40 (36%) in the SPE arm. Other chemotherapy regimens included topotecan monotherapy, amrubicin monotherapy, or other regimens.

### Subset analysis and multivariate analysis

Subset analysis was performed according to PS and age (Table 6). There were no differences in OS between the arms in any subset; thus, an interaction between treatment and PS is unlikely. The survival curves of the patients ⩾70 years of age with a PS of 0–2 are shown in Figure 2C, and the survival curves were very similar with that of original overall populations. Even in the multivariate analysis with seven selected baseline variables, there was no difference in OS between the arms. High lactate dehydrogenase level was most strongly associated with poor prognosis (Table 7).

## DISCUSSION

Until recently, there was no standard chemotherapeutic regimen for elderly SCLC patients. Two phase III (Medical Research Council Lung Cancer Working Party, 1996; Souhami *et al*, 1997) and two randomised phase II trials (Pfeiffer *et al*, 1997; Ardizzoni *et al*, 2005) have shown that suboptimal chemotherapies, such as oral etoposide monotherapy or attenuated doses of combination chemotherapy, may lead to reduced survival in elderly or poor-risk SCLC patients when compared with standard doses of combination chemotherapies. The CE regimen, which has acceptable toxicities and reproducible efficacy, has been used in elderly or poor-risk patients with SCLC worldwide, although there have been substantial differences in toxicities and efficacy between the reported phase II trials. Four trials demonstrated both favourable toxicities and efficacy (Carney, 1995; Evans *et al*, 1995; Matsui *et al*, 1998; Okamoto *et al*, 1999) and three showed somewhat disappointing results because of suboptimal doses of oral etoposide (Larive *et al*, 2002), greater inclusion of patients with poor prognostic factors (Samantas *et al*, 1999), and deterioration of comorbidities as a result of chemotherapy (Quoix *et al*, 2001). No phase III trial evaluating the role of the CE regimen in this population has been reported until now.

This is the first phase III trial comparing carboplatin-based CE and cisplatin-based SPE regimens in elderly or poor-risk patients with ED-SCLC. In addition, this is also the largest randomised trial specifically designed for elderly or poor-risk SCLC patients. Although there was no significant difference in the palliation scores, response rate, and OS between the arms, the efficacy of both regimens was promising, as this study included only elderly or poor-risk patients with SCLC. Most toxicities were tolerable and the treatment compliance was also favourable in both arms. Approximately two-thirds of the patients received all four cycles of treatment. The CE arm in the current trial had more pronounced thrombocytopenia, which was considered manageable because none of the patients in the CE arm showed grade 3 or 4 bleeding, and the CE arm had a slightly prolonged course interval and a slightly greater incidence of dose reduction. However, in our opinion, these toxicities are less meaningful in clinical practice. More importantly, the CE regimen does not require hydration and can be given in an outpatient setting. Based on the results of this study, many JCOG members prefer the CE regimen to the SPE regimen and consider it to be more suitable for the control arm of future phase III trials.

The MST of each regimen (10.6 months for CE *vs* 9.9 months for SPE) was promising considering that this study included only elderly or frail patients with ED-SCLC. However, some retrospective studies have shown that fit elderly patients who have adequate organ functions, a good PS, and no comorbidity are able to tolerate intensive chemotherapy well and show a similar therapeutic response and survival rate as younger patients (Siu *et al*, 1996; Yuen *et al*, 2000). In fact, in this trial the MST of fit elderly patients ⩾70 years of age with a PS of 0–1 was 10.9 months for the CE arm and 10.1 months for the SPE arm. In contrast, the MST of patients with a PS of 3 was only approximately 7 months. Furthermore, the group of fit elderly patients comprised 74% of the patients in this study. Therefore, the favourable survival rates in our trial may be attributable to patient selection. In other words, one limitation of this study is that the results of this trial cannot be extrapolated to frail elderly with a poor PS and/or comorbid illness because of the likelihood of greater inclusion of fit elderly patients in this trial.

Although the total dose in both the CE and SPE arms was slightly lower than the standard regimen, 92% of the patients showed grade 3 or 4 neutropenia, and dose reduction and course delay occurred frequently. However, the MST of both regimens was comparable with that of non-elderly or non-selected patients with ED-SCLC in historical reports (Noda *et al*, 2002; Niell *et al*, 2005). These findings suggest that both regimens are not suboptimal, but are near-full and effective doses for elderly or poor-risk patients with ED-SCLC. The CE arm in the current trial had a slightly prolonged course interval and a slightly greater incidence of dose reduction when compared to the SPE regimen. However, 95% of the patients showed grade 3 or 4 neutropenia and 56% showed grade 3 or 4 thrombocytopenia. Therefore, we believe that the dose escalation of the CE regimen may be difficult in this trial.

It remains unclear whether the elderly are able to tolerate a single modest dose of cisplatin (60–80 mg m^−2^ IV) on day 1. We feel that a fit elderly person who passes strict eligibility criteria can receive a modest dose of cisplatin IV on day 1. However, the more common situation is of elderly patients who have comorbidity and a poor PS, and cannot tolerate a standard single dose of cisplatin. Westeel *et al* (1998) and Murray *et al* (1998) reported that split doses of cisplatin were safely and effectively administered in elderly or frail patients with LD-SCLC. The SPE regimen appeared to be an appropriate treatment for elderly patients with SCLC who cannot tolerate a standard single dose of cisplatin. However, it remains unclear whether fit elderly patients in our trial can tolerate a standard single dose of cisplatin, and if so, it also remains unclear whether fit elderly patients who receive a standard single dose of cisplatin are able to achieve a more improved survival than those who receive SPE. Unfortunately, no randomised study comparing a single standard dose of cisplatin with SPE has been reported in fit elderly patients with SCLC.

There are some problems with the design in this study. The hypothesis was that carboplatin would improve survival, and the design of the trial was a superiority design with survival as the primary end point. However, this hypothesis was based on two possible misconceptions. First, carboplatin could be better dosed and might be more efficacious than cisplatin in SCLC. Unfortunately, this hypothesis could not be sustained on the basis of the available literatures. A number of clinical trials have indicated that carboplatin-based combination chemotherapy has a similar or slightly reduced efficacy compared with cisplatin-based combination chemotherapy against various tumours (Go and Adjei, 1999; Hotta *et al*, 2004). Therefore, our trial should have been designed as a non-inferiority trial. However, if this trial were planned as a non-inferiority trial, a total sample size would be about 500 to 1000 patients, with equal expected survival and a non-inferiority margin for hazard ratio ranging from 1.2 to 1.3. Second, the cisplatin dose in the control arm was an attenuated dose. Souhami *et al* (1997) used reduced dose of cisplatin (60 mg m^−2^ IV on day 1) and Murray *et al* (1998) used a single course of a split cisplatin dose in their studies. These regimens were completely different from the control arm in the present study. A standard dose of cisplatin given in 3 days is the best way of giving standard cisplatin (30 mg m^−2^ IV on days 1–3) with etoposide (130 mg m^−2^ IV on days 1–3), according the North Central Cancer Treatment Group (Maksmiuk *et al*, 1994). Had standard SPE been used for the control arm, better survival might have been achieved with increased toxicities. Another problem with the design was the inclusion of patients with a PS of 3, even if they were less than 70 years old. This made the target population heterogeneous. The number of such patients actually recruited was quite small, so emphasising the inappropriateness of their inclusion. A further limitation of this study may be a long accrual period of five-and-a-half years. Because our oncologists might have been afraid of the risk of TRD or increased toxicities in frail elderly with a poor PS and/or comorbid illness, more fit elderly patients were selectively registered and consequently the accrual rate was very slow.

In our trial, although both regimens were well-tolerated and efficacy was promising, over 90% of the patients in both arms showed grade 3 or 4 neutropenia, which may be justified and acceptable for a clinical trial involving elderly or poor risk patients with ED-SCLC, because only 6% of the patients showed grade 3 or 4 infection and TRD occurred in only four (1.8%) patients. Because all TRD occurred after the first course of chemotherapy, careful monitoring and management is necessary, particularly in the first course, if CE or SPE are administered to elderly or frail patients. Several retrospective analyses (Findlay *et al*, 1991; Radford *et al*, 1992) and a prospective study (Timmer-Bonte *et al*, 2005) have shown that standard-dose chemotherapy without G-CSF support causes more risk of early death and sepsis in the older population. Moreover, the American Society of Clinical Oncology (ASCO) guideline recommends the use of prophylactic G-CSF in patients at higher risk for chemotherapy-induced infection, such as those having a poor PS, older age, or comorbid illness (Smith *et al*, 2006). In this trial, the prophylactic use of G-CSF was recommended, but the actual use was left to the discretion of the treating physician because the use of G-CSF leads to increased drug cost. Although G-CSF was administered in only 54% of the total courses, we believe that the prophylactic use of G-CSF with CE regimen should be recommended in a new trial or clinical practice.

In conclusion, although the SPE regimen is still considered to be the standard treatment for elderly or poor-risk patients with ED-SCLC, the CE regimen can be an alternative for this population considering the risk-benefit balance. Based on the results of our trial, a phase III trial of the CE regimen *vs* amrubicin monotherapy, supported by a pharmaceutical company, is now ongoing in elderly patients with ED-SCLC in Japan, and a comparative trial of the CE regimen *vs* carboplatin plus irinotecan regimen (Okamoto *et al*, 2006) is being discussed for a future trial in our group.

## Figures and Tables

**Table 1:** Patient characteristics

	**CE (****n****=110)**	**SPE (****n****=110)**	***P*-value**
*Age (years)*			
Median (range)	74 (56–86)	73.5 (55–85)	0.34
⩾70 years old (%)	102 (93)	100 (91)	0.81
			
Sex (male/female)	95/15	98/12	0.68
ECOG PS, 0–1/2/3	81/21/8	81/19/10	0.80
⩾5% weight loss	26	38	0.18
			
*LN metastasis*			
Contralateral mediastinum	71	59	0.13
Supraclavicular	89	79	0.15
*Distant metastasis*			
Liver	30	30	1.0
Lung	31	30	1.0
Brain	18	18	1.0
Bone	25	17	0.23
Adrenal	13	7	0.24
Bone marrow	12	12	1.0

CE, carboplatin plus etoposide; ECOG, Eastern Cooperative Oncology Group; LN, lymph node; PS, performance status; SPE, split doses of cisplatin plus etoposide.

**Table 2:** Compliance and drug delivery

	**CE (****n****=110)**	**SPE (****n****=109^a^)**	***P*-value**
Median interval of each chemotherapy (days) (range)			
1–2	27 (14–35)	23 (20–37)	0.02^b^
2–3	25 (21–56)	22 (20–35)	0.07^b^
3–4	27 (21–36)	24 (21–38)	0.05^b^
Total delivered courses/projected courses	353/440 (80%)	360/436 (83%)	
Dose reduction	32 (29%)	11 (10%)	<0.01^c^
Course delay	45 (41%)	40 (37%)	0.58^c^
G-CSF delivery	81 (74%)	84 (77%)	0.64^c^
No. of courses with G-CSF delivery/number of total courses	183/354 (52%)	203/362 (56%)	

CE, carboplatin plus etoposide; G-CSF, granulocyte colony-stimulating factor; SPE, split doses of cisplatin plus etoposide.

a One patient never received chemotherapy due to delirium after registration.

b Wilcoxon rank-sum test.

c Fisher’s exact test.

**Table 3:** Toxicities (JCOG Toxicity Criteria, Worst Grade of Any Course)

	**CE**					**SPE**					
	**Grade**										
**Toxicity**	**1**	**2**	**3**	**4**	**3+4 (%)**	**1**	**2**	**3**	**4**	**3+4 (%)**	***P*-value**
*Haematologic*											
Leucopenia	5	45	46	13	(54)	8	43	49	7	(51)	0.79
Neutropenia	0	5	46	58	(95)	4	7	41	57	(90)	0.22
Anaemia	9	58	32	—	(29)	20	45	27	—	(25)	0.54
Thrombocytopenia	20	18	29	32	(56)	16	15	12	5	(16)	<.01
											
*Non-haematologic*											
Nausea/vomiting	40	24	2	—	(2)	46	28	3	—	(3)	0.68
Diarrhoea	8	9	1	0	(1)	11	3	1	0	(1)	1.0
Bilirubin	—	31	0	0	(0)	—	16	1	0	(1)	0.50
AST	47	9	3	0	(3)	30	8	6	0	(6)	0.33
ALT	40	9	2	0	(2)	38	8	4	0	(4)	0.45
Creatinine	10	2	0	0	(0)	27	3	1	0	(1)	0.50
Hyponatraemia	38	11	7	11	(16)	46	20	6	9	(14)	0.58
PaO2	39	21	7	1	(10)	44	23	2	1	(4)	0.22
Fever	15	15	0	0	(0)	21	16	0	0	(0)	—
Infection	12	15	5	3	(7)	16	7	5	1	(6)	0.78
Bleeding	8	1	0	0	(0)	4	0	0	0	(0)	—
Neurologic-sensory	2	1	0	—	(0)	3	2	0	—	(0)	—
Alopaecia	67	22	—	—		66	15	—	—		

CE, carboplatin plus etoposide; JCOG, Japan Clinical Oncology Group; PaO_2_, partial pressure of oxygen; SPE, split doses of cisplatin plus etoposide.

**Table 4:** Palliation score

	**CE**		**SPE**		
	**Change from baseline**		**Change from baseline**		
**Symptom**	**Mean (s.d.)**	**Median (range)**	**Mean (s.d.)**	**Median (range)**	******P**** a**
Cough	−0.38 (1.16)	0 (−3 to 3)	−0.54 (1.06)	0 (−3 to 3)	0.51
Pain	−0.19 (1.00)	0 (−3 to 3)	−0.19 (0.96)	0 (−3 to 3)	0.96
Anorexia	−0.07 (1.16)	0 (−3 to 3)	0.08 (1.22)	0 (−3 to 3)	0.37
Shortness of					
breath	−0.05 (1.02)	0 (−2 to 3)	−0.31 (0.95)	0 (−3 to 3)	0.12
Well-being	−0.15 (1.13)	0 (−3 to 3)	−0.02 (1.14)	0 (−3 to 3)	0.48
Nausea	0.16 (0.84)	0 (−2 to 3)	0.26 (0.80)	0 (−1 to 3)	0.21
Diarrhoea or					
constipation	0.05 (1.07)	0 (−3 to 3)	0.04 (0.99)	0 (−3 to 3)	0.69
Sleep	−0.15 (1.08)	0 (−3 to 3)	−0.04 (0.89)	0 (−3 to 2)	0.10
					
Total	−0.80 (6.04)	−2 (−12 to 22)	−0.71 (5.35)	−1 (−15 to 21)	0.32

CE, carboplatin plus etoposide; s.d., standard deviation; SPE, split doses of cisplatin plus etoposide.

a Wilcoxon rank-sum test.

**Table 5:** Therapeutic response (WHO)

	**CE**	**SPE**	**Total**
CR	5	5	10
PR	75	75	150
NC	17	11	28
PD	11	16	27
NE	2	3	5
			
Total	110	110	220
			
Response rate	73%	73%	
95% CI	63–81%	63–81%	

CE, carboplatin plus etoposide; CI, confidence interval; CR, complete response; NC, no change; NE, not evaluable; PD, progressive disease; PR, partial response; SPE, split doses of cisplatin plus etoposide; WHO, World Health Organization.

**Table 6:** Subset analysis – overall survival

		**MST (months)**	
**Subgroup**	**Number of patients (%)**	**CE**	**SPE**
PS 0–1	162 (74)	10.9	10.1
PS 2–3	58 (26)	8.3	8.1
<70 years and PS 3	18 (8)	7.1	6.9
⩾70 years and PS 0–2	202 (92)	10.8	10.0

CE, carboplatin plus etoposide; MST, median survival time; PS, performance status; SPE, split doses of cisplatin plus etoposide.

**Table 7:** Multivariate analysis with baseline prognostic factors

**Variables**	******P****-value**	**Hazard ratio**	**95% CI**
Treatment arm (CE vs. SPE)	0.99	0.99	0.75–1.33
Alkaline phosphatase level (normal *vs* abnormal)	0.97	0.99	0.68–1.46
Lactate dehydrogenase level	<0.001	1.69	1.23–2.26
(⩾ × 1.5 *vs* < × 1.5)			
Leucocyte count			
(⩾10 000/mm^3^ *vs* <10 000/mm^3^)	0.06	1.82	0.99–3.36
Age (⩾75 years *vs* <75 years)	0.77	1.05	0.78–1.41
PS (2–3 *vs* 0–1)	0.41	1.15	0.82–1.61
Sex (female *vs* male)	0.13	0.70	0.45–1.11

CE=carboplatin plus etoposide; SPE=split doses of cisplatin plus etoposide; PS=performance status; CI=confidence interval.

## Appendix

This study was coordinated by the Japan Clinical Oncology Group (N Saijo, Chairperson) and was performed with the cooperation of the following institutions and investigators: Tochigi Cancer Center Hospital, Tochigi (K Mori, M Noda, T Kondo, and Y Kamiyama); National Nishi-Gunma Hospital, Gunma (S Tsuchiya, Y Koike, K Satoh, A Tohi, and K Kaira); Gunma Cancer Center Hospital, Gunma (K Minato); Saitama Cancer Center Hospital, Saitama (H Sakai, K Kobayashi, and R Kuroki); National Cancer Center, Central Hospital, Tokyo (T Tamura, Y Ohe, H Kunitoh, I Sekine, H Nokihara, and H Murakami); National Cancer Center Hospital East, Chiba (R Kakinuma, K Kubota, H Ohmatsu, K Gotoh, and S Niho); National International Medical Center, Tokyo (Y Takeda, S Izumi, A Kawana, M Kamimura, and M Iikura); Toranomon Hospital, Tokyo (K Kishi, and M Kawabata); Kanagawa Cancer Center Hospital, Kanagawa (K Yamada, I Nomura, F Oshita, and M Ikehara), Yokohama Municipal Citizen's Hospital, Kanagawa (K Watanabe, H Kunikane, H Okamoto, A Nagatomo, and H Aono); Niigata Cancer Center Hospital, Niigata (A Yokoyama, H Tsukada, M Makino, T Shinbo, S Kinebuchi, J Tanaka, M Tango, and T Ohara); Gifu City Hospital, Gifu (T Sawa, M Miwa, T Ishiguro, M Sawada, and T Yoshida); Aichi Cancer Center Central Hospital, Aichi (K Yoshida, and T Hida); Aichi Cancer Center Aichi Hospital, Aichi (H Saitoh, and M Okuno); Osaka City University Medical School, Osaka (S Kudoh, S Kyoh, H Kamoi, N Yoshimura, T Kodama, K Ohtani, S Shiraishi, S Nomura, S Enomoto, H Matsuura, and R Wake); Kinki University Medical School, Osaka (T Nogami, N Yamamoto, S Sakai, K Kodama, K Akiyama, J Tsurutani, K Tamura); Osaka Prefectural Adult Disease Center, Osaka (S Nakamura, F Imamura, M Yoshimura, S Yamamoto, K Ueno, H Ohmiya, H Matsuoka, and H Uda); Osaka Prefectural Respiratory and Allergy Medical Center, Osaka (M Furukawa, T Yamadori, T Takimoto, and T Hirashima); National Kinki Central Thoracic Disease Center, Osaka (S Minami, N Naka, T Kawaguchi, and H Ishikawa); National Toneyama Hospital, Osaka (Y Okano); Osaka City General Medical Center, Osaka (N Takifuji, and M Miyazaki); Kobe City Central Hospital, Kobe (T Nishimura, Y Okazaki, D Kinose, H Fujii, S Takakura, and M Hayashi); Sasebo City General Hospital, Nagasaki (J Araki); Kumamoto Regional Medical Center, Kumamoto (H Senba, T Seto, and S Fujii).
